# First case of bloodstream infection caused by NDM-positive *Escherichia hermannii*

**DOI:** 10.1186/s12879-023-08336-3

**Published:** 2023-05-25

**Authors:** Bin Lu, Bin Wang, Xinling Pan, Chenxin Liu, Chenyuan Jin, Yunzhen Shi, Yangxiao Zhou

**Affiliations:** 1 Department of Infectious Diseases, Affiliated Dongyang Hospital of Wenzhou Medical University, No. 60 Wuningxi Road, Dongyang, Zhejiang Province China; 2 Department of Emergency, Affiliated Dongyang Hospital of Wenzhou Medical University, Dongyang, Zhejiang Province China; 3 Department of Biomedical Sciences Laboratory, Affiliated Dongyang Hospital of Wenzhou Medical University, Dongyang, China; 4 Department of Clinical Laboratory, Affiliated Dongyang Hospital of Wenzhou Medical University, No. 60 Wuningxi Road, Dongyang, Zhejiang China

**Keywords:** *Escherichia hermannii*, NDM-positive, Bloodstream infection, Aztreonam

## Abstract

**Background:**

*Escherichia hermannii (E. hermanni)* is always accompanied by other bacterial infections in humans. In previous reports, most *E. hermannii*-related infections were caused by sensitive strains. Here, for the first time, we report the case of a patient with New Delhi metallo-β-lactamase (NDM)-positive *E. hermannii* bloodstream infection.

**Case presentation:**

The patient was a 70-year-old male admitted to our hospital due to a 4-day fever, with a history of malignant tumor, liver cirrhosis, and chronic obstructive pulmonary disease. After admission, his blood culture tested positive for *E. hermannii*. The drug resistance analysis showed positive for NDM resistance, with susceptibility to aztreonam, levofloxacin, and amikacin. The blood culture turned negative after 8 days of aztreonam treatment. The patient’s symptoms improved, and he was discharged after 14 days of hospitalization.

**Conclusions:**

This is the first report of a bloodstream infection caused by an NDM-positive *E. hermannii* strain. The anti-infection regimen used in this case provides a new reference regimen for clinical practice.

## Background

*Escherichia hermannii* (*E. hermannii*) is a gram-negative and facultative anaerobic bacterium first reported in 1982. It is a member of the *Enterobacteriaceae* family and was reclassified as a distinct species within the *Escherichia* genus based on the biochemical and genomic differences from *Escherichia coli* [1]. In recent years, *E. hermannii* has been found as a single causative agent of infection [2–4]. Most *E. hermannii* strains are antibiotic sensitive, while a few are resistant to antibacterial drugs such as penicillins, cephalosporins, quinolones and carbapenems [5, 6]. In this study, we report a bloodstream infection case caused by a New Delhi metallo-β-lactamase (NDM)-positive *E. hermannii* strain. This strain was resistant to penicillin, cephalosporins, β-lactamase inhibitor combinations, and carbapenems. The patient improved after aztreonam treatment. Practitioners must pay close attention to infections caused by NDM positive *E. hermannii*, particularly for immunodeficient populations.

## Case presentation

A 70-year-old male patient was admitted to Affiliated Dongyang Hospital of Wenzhou Medical University on July 23, 2022, after experiencing chills and fever for 4 days. The physical examination upon admission revealed the following vital signs: temperature 39.5 °C, heart rate 82 beats/min, respiratory rate 20 beats/min, blood pressure 149/99 mmHg. The abdomen was distended without tenderness and rebound pain and with positive shifting dullness.

The patient had a history of hypertension, hepatitis B cirrhosis, and chronic obstructive pulmonary disease. Six months before admission, the patient underwent total gastrectomy due to a gastric malignant tumor diagnosis with a secondary malignant tumor in the abdominal cavity, followed by two chemotherapies after surgery. In addition, he presented with obstructive hydronephrosis 23 days before admission, and a double J catheter was placed in another hospital. He was then hospitalized in our facilities due to anuria 19 days before admission, where he underwent a nephrostomy for both kidneys and was discharged after hemodialysis treatment.

## Laboratory examinations at admission

**Fig. 1:**
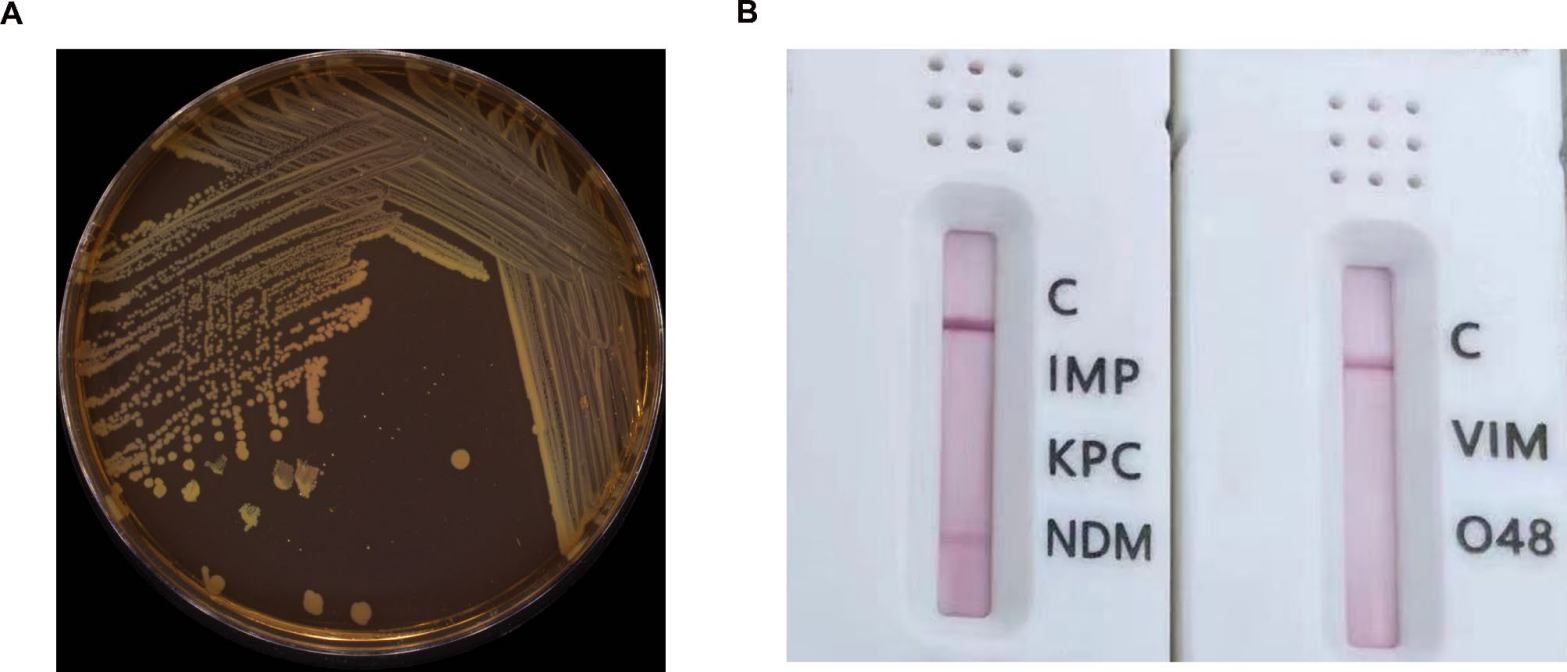
Identification of an NDM-positive *Escherichia hermannii.* (**A**) The colony morphology of *Escherichia hermannii* on the blood culture plate; (**B**) The enzymes for carbapenem resistance by gold immunolabeling test

A blood sample was collected immediately after hospitalization and sent for bacterial culture within 2 h. After incubation on a blood-agar plate at 37℃ for 48 h, only one bacterial morphology type formed (Fig. 1A). The species was identified as *E. hermannii* by mass spectrometry. The drug susceptibility test was performed by using AST-335 card and AST334 card on a VITEK® 2 Compact system (VITEK, France) and minimum inhibitory concentration was read automatically (Table 1). This strain was resistant to penicillin, cephalosporins, β-lactamase inhibitor combinations (cefoperazone /sulbactam, piperacillin/ tazobactam, amoxicillin /clavulanate and ticarcillin /clavulanate), carbapenems, but sensitive to aztreonam, tigecycline, levofloxacin, and amikacin. Gold immunolabeling test (Mountainriver, Beijing, China) showed that this strain was positive for NDM (Fig. 1B), negative for KPC, O48 and VIM. The blood routine test showed several abnormal indexes: 9.19 × 10^9/L white blood cells (WBCs), 94.4% neutrophils, 79 g/L hemoglobin, 206.33 mg/L hypersensitive C-reactive protein (CRP), 2.96 ng/ml procalcitonin (PCT). A positive WBCs result and abnormal urea (10.1 mmol/L) and creatinine (156 µmol/L) levels were found in urine. An abdominocentesis was performed 3 days after admission showing turbid yellow ascites. The Rivalta test was positive, revealing 1.195 × 10^9/L nucleated cells, where multinucleated cells accounted for 74.3% and mononuclear for 25.7%. Ascites and urine were both negative in culture examinations.

**Table 1:** The minimum inhibitory concentrations of an NDM-positive *Escherichia hermannii*

Antibiotic agent	MIC (µg/ml)	phenotype
Aztreonam	<=1	S
Amikacin	<=2	S
Cefoperazone/sulbactam	>=64	R
Piperacillin/tazobactam	>=128	R
Amoxicillin/clavulanate	>=32	R
Ticarcillin/clavulanate	>=128	R
Ceftriaxone	>=64	R
Ceftazidime	>=64	R
Cefuroxime	>=64	R
Cefoxitin	>=64	R
Imipenem	>=16	R
Meropenem	>=16	R
Levofloxacin	0.5	S
Tigecycline	<=0.5	S
Sulfamethoxazole	>=320	R

## Diagnosis and treatments

**Fig. 2:**
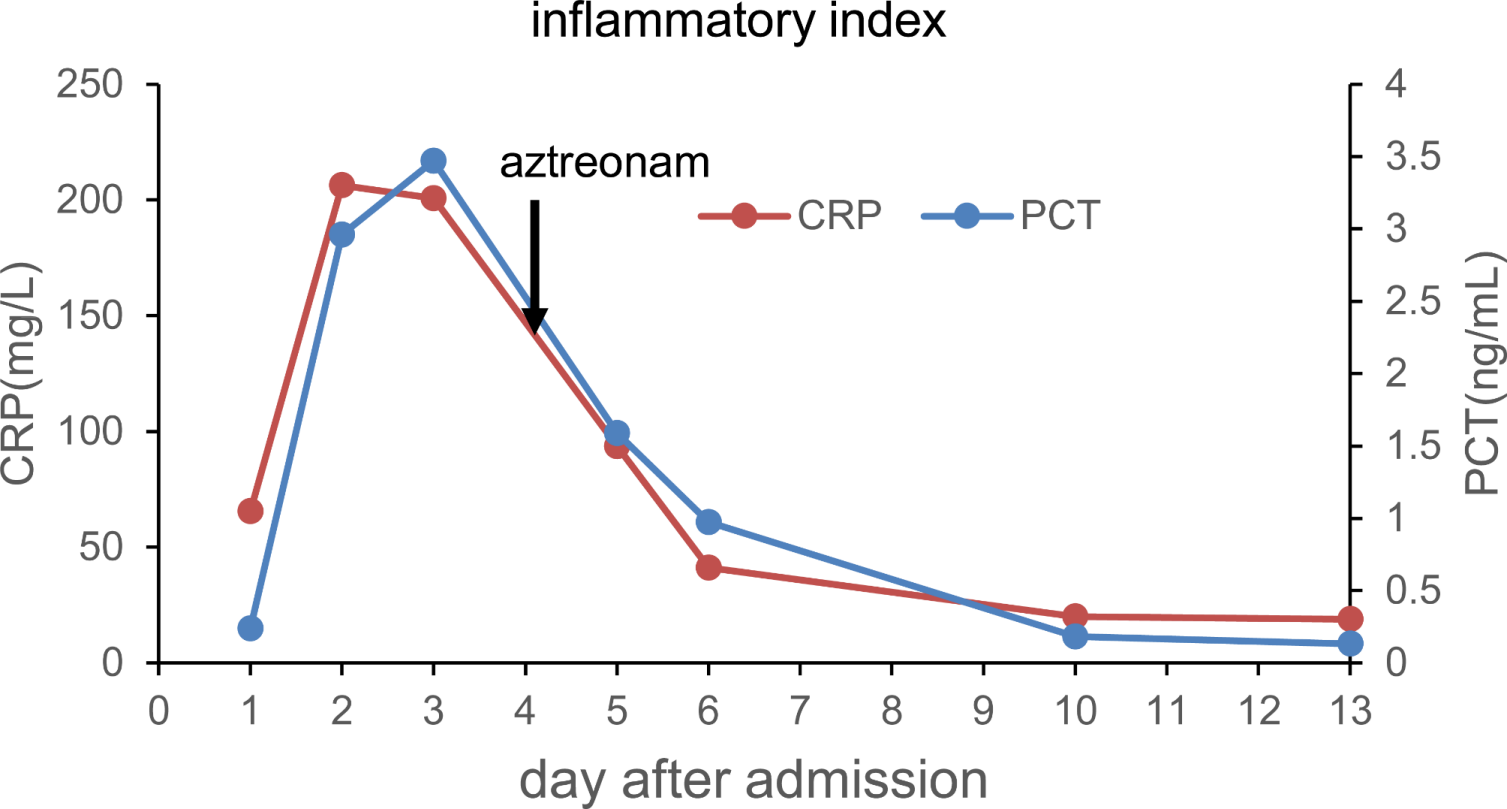
The inflammatory indexes of this case infected by NDM-positive *Escherichia hermannii* during hospitalization

On the first day after admission, the patient was treated with 2 g intravenous cefoperazone-sulbactam every 12 h and acetaminophen for defervescence and fluid replacement. On the 3rd day after admission, the patient’s blood inflammatory indicators were not improving (PCT 3.47 ng/ml; CRP 200.83 mg/L). According to the species identification and drug susceptibility results, the antibiotic treatment was switched to 1 g intravenous aztreonam every 8 h on the 4th day after admission. Consequently, blood inflammation indicators gradually decreased (Fig. 2), and on the 8th day of treatment, the blood culture turned negative. The patient’s symptoms improved, and he was discharged after 14 days of hospitalization.

## Discussion and conclusions

*E. hermannii* was initially proposed as a new species by Brenner in 1982. It has been successfully isolated from several infection sites, including wounds [7], peritonitis [8], conjunctivitis [9], blood [10], cerebrospinal fluid [11], and urine [12]. The most common infection sites comprise bloodstream, urinary tract, and central nervous system infections [5]. In this report, we described a case with bloodstream infection caused by *E. hermanii*. Recently, previous studies have reported *E. hermannii* as the only causative agent, including patients with open tibial shaft fractures [2], uremia requiring long-term hemodialysis [3], and after peripherally inserted central catheter placement [4]. The patient had underlying diseases such as a malignant tumor, hepatitis B cirrhosis, and chronic obstructive pulmonary disease, indicating he was immunocompromised. Moreover, the patient was submitted to ureteral catheter placement and nephrostomy, including both kidneys, within one month before admission, which increased the risk of *E. hermannii* infection. No other bacterial or fungal growth was detected in the blood culture, with negative ascites and urine cultures. Therefore, *E. hermannii* was the only pathogen causing the bloodstream infection.

NDM resistance was initially identified in 2009 in *Klebsiella pneumoniae* isolated from an Indian-Swedish patient’s urine after a urinary tract infection in New Delhi, India [13]. Since this first isolation, NDM-resistant strains have been isolated for *Acinetobacter baumannii*, *Escherichia coli*, *Salmonella*, and other bacteria [14–16]. Our case is the first report on a bloodstream infection caused by NDM-positive *E. hermannii*.

Antibiotic selection for infectious disease treatment is primarily based on drug susceptibility results. Several previous reports show that *E. hermannii* is sensitive to most antibacterial drugs [5, 17]. However, a previous case by Sood et al. [6] detailed a bloodstream infection caused by *E. hermannii* in a 2-day-old infant. The strain was resistant to penicillin, cephalosporins, and carbapenems and sensitive to polymyxin and tigecycline. The patient recovered after 2 weeks of combined anti-infection treatment with polymyxin and amikacin. The NDM-positive *E. hermannii* strain in this work was resistant to penicillin, cephalosporins, β-lactamase inhibitor combinations, and carbapenems, but sensitive to aztreonam, levofloxacin, amikacin, and tigecycline. Since there was no previous experience for NDM-positive *E. hermannii* infection therapy, we decided to treat with aztreonam according to in vitro drug susceptibility test results. After 8 days of treatment, the blood culture turned negative and inflammatory index descreased, and after 14 days, the patient recovered from the infection and was discharged.

The patient was admitted to the hospital again 11 days after discharge due to other symptoms. Although his family members decided to discontinue treatment because of the patient’s cancer terminal stage, the treatment of *E. hermannii* bloodstream infection was successful. This is the first case with effective treatment for an NDM-positive *E. hermannii* infection, providing insight into optional solutions for the treatment of similar future conditions.

Bloodstream infections caused by *E. hermannii* are clinically rare and usually present in patients with underlying immunodeficiency diseases. Most of them are caused by susceptible strains. This is the first report of a bloodstream infection caused by an NDM-positive *E. hermannii* strain. We formulated a specific antibacterial drug treatment plan based on in vitro drug susceptibility test results. The anti-infection regimen used in this case provides a new reference regimen for clinical practice.

## Data Availability

The datasets used during the current study are available from the corresponding author on reasonable request.

## Abbreviations

*E. hermannii*: *Escherichia hermannii*
NDM: New Delhi metallo-β-lactamase
WBCs: White blood cells
